# Association between substance use and PrEP adherence among adolescent girls and young women enrolled in an HIV prevention study in Southern Africa

**DOI:** 10.1371/journal.pgph.0004750

**Published:** 2025-06-18

**Authors:** Kudzai Hlahla, Rahul Paul Choudhury, Bekezela Siziba, Peter L. Anderson, Sinead Delany-Moretlwe, Theodorah Rirhandzu Ndzhukule, Sybil Hosek, Nyaradzo M. Mgodi, for the on behalf of the HPTN 082 Study Team

**Affiliations:** 1 Clinical Trials Research Centre, University of Zimbabwe Faculty of Medicine and Health Sciences, Harare, Zimbabwe; 2 HPTN SDMC Statistical Center for HIV/AIDS Research & Prevention (SCHARP), Seattle, Washington, United States of America; 3 University of Colorado Anschutz Medical Campus, Aurora, Colorado, United States of America; 4 Wits Reproductive Health and HIV Institute (Wits RHI), Johannesburg, South Africa; 5 Desmond Tutu Health Foundation, Cape Town, South Africa; 6 HIV Prevention Trials Network 082 (HPTN 082) Protocol Team, Durham, North Carolina, United States of America; The Chinese University of Hong Kong, HONG KONG

## Abstract

Adolescent girls and young women (AGYW) in sub-Saharan Africa are at substantial risk of HIV acquisition and could benefit from oral pre-exposure prophylaxis (PrEP) for HIV prevention. Substance use may result in poor adherence, diminishing PrEP effectiveness. The effects of substance use on PrEP adherence in AGYW within the African context have not been extensively studied. We sought to determine the prevalence of substance use and its association with PrEP adherence in AGYW enrolled in an HIV prevention trial. The HIV Prevention Trials Network (HPTN) 082 study enrolled healthy, HIV-negative, sexually active young women (16–25 years) from Cape Town, Johannesburg, South Africa, and Harare, Zimbabwe, between October 2016–2018. Participants were offered oral PrEP. Data on hazardous drinking was collected using the AUDIT-C questionnaire and defined as having an AUDIT-C score ≥3. Data on substance use was collected using the abridged ASSIST questionnaire, with responses categorized as 0 (never used a substance) and 1 (ever substance use). Tenofovir-diphosphate (TFV-DP) levels in dried blood spots at weeks 13, 26, and 52 were used to measure PrEP adherence. Low adherence was defined as TFV-DP concentration <700 fmol/punch. Repeated measure multinomial regression modeling was used to determine associations between substance use and hazardous drinking vs PrEP adherence. Of the 451 participants enrolled, 427 (95%) initiated PrEP. The prevalence of hazardous drinking and substance use at baseline was 37% and 24%, respectively. Hazardous drinking was highest in Cape Town (53%). Substance use was highest in Johannesburg (31%). Cannabis (7%) and sedatives (6%) were the most frequently used substances. Hazardous drinking (aOR=1.55, 95%CI = 1.05-2.29) was associated with increasing odds of low PrEP adherence. Substance use was not associated with low PrEP adherence. There is a need for increased screening for hazardous drinking and substance use and its integration within PrEP programs in AGYW in Africa.

## Introduction

According to UNAIDS, in 2022, 210,000 adolescent girls and young women (AGYW), between the ages of 15 and 24, were diagnosed with HIV globally, with the greatest incidence being in Eastern and Southern Africa [1]. Six in seven HIV infections among adolescents in sub-Saharan Africa (SSA) occurred in girls, with AGYW being three times more likely to become HIV-positive compared to their age-matched male counterparts [2,3]. The HIV incident rate in Zimbabwe among AGYW is three times more than that in young men; in South Africa, 35% of all new infections occur among AGYW [4,5]. Increased vulnerability in this population has been attributed to various socio-economic factors, including gender inequality, violence, poverty, stigma and discrimination surrounding sexuality, and lack of access to sexual reproductive health care education and services such as pre-exposure prophylaxis (PrEP) [2,6]. The provision of oral PrEP in the form of tenofovir and emtricitabine (TDF/FTC), which is a safe and effective HIV prevention tool, has therefore been prioritized for use among AGYW in southern African countries like South Africa and Zimbabwe [7–11]. However, randomized controlled trials amongst young African women have shown PrEP non-efficacy, mostly due to PrEP non-adherence in this population [12–14]. It is therefore imperative to identify and address potential barriers to PrEP use in AGYW in Africa.

Hazardous substance use, which is defined as the harmful and continued use of alcohol, illegal drugs, or pharmaceuticals (including over-the-counter medications) for non-prescription purposes, is a growing global public health concern amongst adolescents and young people in the region [15,16]. Alcohol and substance use in AGYW specifically has been on the increase despite the social and cultural beliefs surrounding the use of substances by women in the African setting [17]. Alcohol is reported to be the most commonly used psychoactive substance in Africa, with systematic reviews showing a prevalence of alcohol use by adolescents in Southern Africa ranging from 23.1 to 40.82% [18–21]. South Africa is reported to have a higher prevalence of hazardous drinking, with population-based studies showing a prevalence of 19% compared to 4.5% in Zimbabwe [22,23]. The prevalence of substance use in Southern Africa specifically is estimated to be 37%, with cannabis being the most commonly used substance in both Zimbabwe and South Africa [24]. However, emerging data is showing an increase in the use of stimulants such as cocaine and methamphetamine, opiates, prescription medicines, and cough syrups by adolescents under the age of 19 years in both countries [25,26].

Substance use during adolescence and young adulthood is thought to cause reduced cognitive function and decision-making capacity, resulting in increased risky sexual behavior and the inability to utilize HIV prevention tools such as condoms and PrEP [15,27,28]. Studies among gay, bisexual, and other men who have sex with men have shown that substance use may result in decreased PrEP adherence or PrEP discontinuation [28,29]. However, to our knowledge, there is no published data examining the impact that alcohol and substance use has on PrEP uptake and adherence among AGYW in Southern Africa.

Data on the nature, extent, and potential impact of substance use in AGYW in the African context could be critical for informing the successful design and rollout of PrEP programs and policies in this sub-population. Therefore, to fill this knowledge gap, we aimed to determine the prevalence of substance use and hazardous drinking, and their associations with PrEP uptake and persistence, among AGYW at risk of HIV acquisition in Zimbabwe and South Africa.

## Methods

### Ethical statement

Ethical approvals for the parent protocol were obtained from the University of Cape Town Faculty of Health Sciences (reference number: 129/2016), the University of Witwatersrand, Human Research Ethics Committee (reference number: 160304), and the University of Zimbabwe Joint Research Ethics Committee (reference Number: 27/16). All participants provided written informed consent in English or their local language of choice. Participants retained a signed copy of the informed consent form. Following local regulations, participants below the legal age for consent provided assent, and parents or guardians provided informed consent [30]. All procedures and data analyzed in this sub-analysis were collected in the parent protocol and approved by the parent protocol ethics approval.

### Study design and setting

This analysis uses data from a prospective randomized controlled study conducted by the HIV Prevention Trials Network (HPTN), called HPTN 082 [ClinicalTrials.gov NCT02732730]. HPTN 082 evaluated PrEP uptake, persistence, and the effect of drug level feedback on PrEP adherence among AGYW in three study sites in Harare, Zimbabwe, Cape Town, and Johannesburg South Africa [30]. The HPTN 082 study enrolled 451 sexually active, HIV-negative young women aged 16–25 years, who were at risk of HIV according to the Vaginal and Oral Interventions to Control the Epidemic (VOICE) risk score from October 2016 to October 2018 [30]. The VOICE risk score is a validated tool that uses a point system to screen and identify women at risk of HIV acquisition in Eastern and Southern Africa [31]. Enrolled participants were offered PrEP and randomized to receive either standard adherence support which included adherence counseling, 2-way SMS that allowed participants to receive and send messages thereby enabling them to respond, and adherence clubs. Participants could also be randomized to the enhanced adherence support arm, which had all components of the standard adherence support but with added drug-level feedback from dried blood spot (DBS) concentrations of intracellular tenofovir-diphosphate (TFV-DP). Participants were followed up for 12 months and tested for HIV and pregnancy at every visit. The results of the HPTN 082 study showed a decrease in PrEP adherence with continued study participation as measured by TFV-DP DBS levels, and no difference in PrEP adherence when comparing standard adherence support versus enhanced adherence support [30]. In this paper, we evaluated the role of substance use and hazardous drinking on PrEP adherence among participants enrolled in HPTN 082 who completed at least one follow-up visit.

### Study procedures and data collection

AGYW who consented to HPTN 082, and initiated PrEP received a month’s supply of oral TDF/FTC at enrolment and monthly for the first three months, then quarterly thereafter. DBS samples for storage were collected at study visits where oral PrEP was dispensed, including the exit visit. DBS samples were used to measure intracellular TFV-DP concentrations (fmol/punch). Intracellular TFV-DP concentrations at weeks 13, 26, and 52 were used as an indicator of PrEP adherence. As per the main study, low adherence, which does not offer optimal HIV prevention protection, was defined as having TFV-DP concentration <700 fmol/punch (which ranged from below the limit of quantification to 699 fmol/punch), indicative of taking 3 or fewer tablets per week on average [30].

At screening and subsequent visits where women received oral PrEP, standardized Computer-Assisted Self-Interview (CASI) questionnaires were completed in a language of their choice, where data on participant characteristics, including socioeconomic variables, sexual behaviors, mental health, and alcohol and drug use, were collected. Data on hazardous drinking was collected using the Alcohol Use Disorder Identification Test-Concise (AUDIT-C) questionnaire [32]. The AUDIT-C is a validated brief version of the AUDIT questionnaire that is used to identify hazardous drinking or alcohol use disorders in an individual. The questionnaire comprises three questions, with each response scoring between 0–4 points. The complete questionnaire can be scored on a scale of 0–12. Hazardous drinking is defined as having an AUDIT-C score ≥3 in women, in line with National Institute of Health and National Institute of Drug Abuse (NIDA) guidelines [32].

The frequency of use of different illicit substances was collected using one question of the Alcohol, Smoking and Substance Involvement Screening Test (ASSIST) questionnaire [33]. Participants were asked to indicate how often they had used cannabis, cocaine, amphetamine-type stimulants, inhalants, sedatives/sleeping pills, hallucinogens, opioids, prescription drugs for non-prescription purposes, or any other substance in the past month. Responses were scored between 0 (never used a substance) and 4 (daily use of substance). In addition, participants were also asked in a separate question if they had ever used a needle to inject a drug.

### Exposure and outcome variables

Hazardous drinking was defined as having an AUDIT-C score ≥3, while substance use was classified as the use of any substances listed in the questionnaire in the past month. Substance use was categorized as none (score = 0) and ever substance use (score = 1) based on the responses from the frequency of substance use reported.

The outcome variable was PrEP adherence, measured by TFV-DP concentrations in DBS samples. PrEP drug concentration was categorized into 3 categories: i) BLQ – 349 fmol/punch (fewer than two tablets a week), ii) 350 –699 fmol/punch (two to three tablets a week), and iii) >700 fmol/punch (four or more tablets a week). Low PrEP adherence was defined as having intracellular TFV-DP concentration <700 fmol/punch. Other explanatory variables included age, site, and educational level. Age was divided into three categories: 16–17 years, 18–21 years, and 22–26 years, per parent protocol. Sites were categorized as Harare, Cape Town, and Johannesburg. Lastly, education level was categorized into four categories, namely ‘no schooling’, ‘primary school’, ‘secondary school’, and ‘college or university’.

### Data analysis

Data analysis was conducted using SAS v.9.4. The data collected is available for public viewing on Dryad [34]. The prevalence of alcohol and substance use was analyzed for all participants who initiated PrEP. In addition, the prevalence of polysubstance use and the use of a needle to inject a substance was also determined. To further understand the association between substance use, hazardous drinking, and PrEP adherence, we compared the mean TFV-DP concentrations against AUDIT-C and ever substance use by site and by visit. We used a repeated measures multinomial regression model to determine the association between substance use and hazardous drinking, and with PrEP adherence over time. Independent variables were hazardous drinking, as defined by the AUDIT-C score, and substance use as defined by the ever use of substances listed in the CASI. The reference for all the independent variables was the lowest values (<3 for the AUDIT-C score and never for substance use). The outcome variable was PrEP adherence, measured by TFV-DP concentrations in DBS samples. PrEP drug concentration was categorized into 3 categories: i) BLQ – 349 fmol/punch (fewer than two tablets a week), ii) 350 –699 fmol/punch (two to three tablets a week), and iii) >700 fmol/punch (four or more tablets a week). Low PrEP adherence was defined as having intracellular TFV-DP concentration <700 fmol/punch. The model was adjusted for site, age, and education level as these covariates could affect PrEP adherence. The model provided unadjusted and adjusted odds ratios and confidence intervals.

## Results

### Sociodemographic characteristics of PrEP acceptors

**Fig 1:**
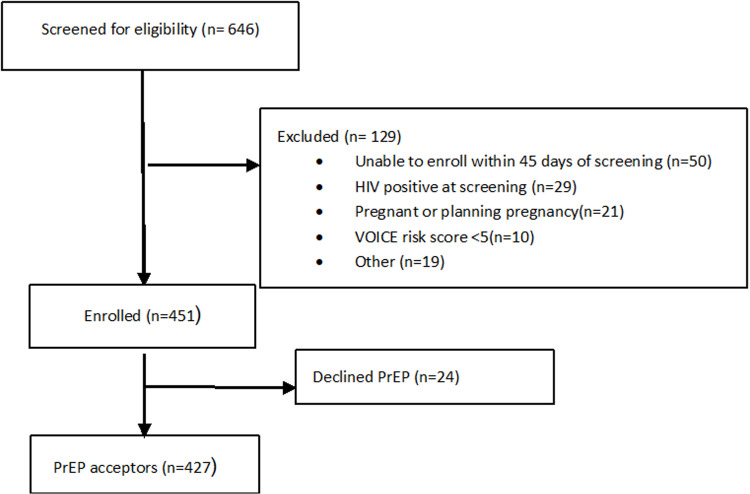
Participant Eligibility Flow Chart.

A total of 451 AGYW were enrolled in the study, and 427 (95%) initiated PrEP (Fig 1). Participant characteristics are shown in Table 1. The majority (56.0%) of the AGYW were 18–21 years of age and had a secondary school education (86.9%). The distribution of participants across sites was similar, with the Johannesburg site enrolling 142 (33.2%) acceptors whilst the Cape Town site enrolled 140 (32.8%). The Zimbabwe site had 145 (34.0%) PrEP acceptors.

**Table 1:** Baseline demographics of PrEP acceptors by site.

	Overall	Harare	Cape Town	Johannesburg
**Total Participants Enrolled**	427	145 (34.0%)	140 (32.8%)	142 (33.2%)
**Age (years)**				
16 – 17	27 (6.3%)	10 (6.9%)	15(10.7%)	2 (1.4%)
18 – 21	239 (56.0%)	72 (49.7%)	98 (70.0%)	69 (48.6%)
22 – 26	161 (37.7%)	63 (43.4%)	27 (19.3%)	71 (50.0%)
**Education**				
No Schooling	0 (0.0%)	0 (0.0%)	0 (0.0%)	0 (0.0%)
Primary School	9 (2.1%)	7 (4.8%)	1 (0.7%)	1 (0.7%)
Secondary School	371 (86.9%)	136 (93.8%)	126 (90.0%)	109 (76.8%)
College or University	47 (11.0%)	2(1.4%)	13 (9.3%)	32(22.5%)
Participant currently in school	161 (37.7%)	8 (5.5%)	63 (45.0%)	90 (63.4%)
Participant ever dropped out of school	125 (29.3%)	58 (40.0%)	45 (32.1%)	22 (15.5%)

### Baseline alcohol and substance use of PrEP acceptors

At baseline, over half of the women (57%) reported ever consuming alcohol, while one-third (37%) reported never having had alcohol (Table 2). Reported alcohol use was higher at the two South African sites, with 74% and 69% of women at the Cape Town and Johannesburg sites reporting alcohol use, respectively, compared to 30% at the Harare site. Overall, a third (37%, 95% CI [33%, 42%]) of young women had an AUDIT-C score greater than or equal to 3 and were classified as hazardous drinkers. Hazardous drinking was most prevalent among young women from the Cape Town site (53%), followed by the Johannesburg site (44%), and the Harare site (16%).

**Table 2:** Baseline proportions of alcohol and substance use of PrEP acceptors by Site.

	Overall (N = 427) [(95% CI])^*^	Harare (N = 145)	Cape Town (N = 140)	Johannesburg (N = 142)
**How often do you have a drink containing alcohol (including Zed)?**				
Never	158 (37.0%)	95 (66.0%)	24 (17.0%)	39 (27.0%)
Monthly or less	132 (31.0%)	26 (18.0%)	49 (35.0%)	57 (40.0%)
2 to 4 times a month	87 (20.0%) (0.15, 0.22)	11 (8.0%)	41 (29.0%)	35 (25.0%)
2 to 3 times a week	23 (5.0%) (0.03, 0.07)	4(3.0%)	13 (9.0%)	6 (4.0%)
4 or more times a week	3 (1.0%) (0.00, 0.01)	1(1.0%)	2 (1.0%)	0 (0.0%)
Prefer not to answer	22 (5.0%)	8(6.0%)	10 (7.0%)	4 (3.0%)
Missing	2 (<1.0%)	0 (0.0%)	1 (1.0%)	1 (1.0%)
**Audit Score**				
<3 (Nonhazardous drinking)	259 (60.7%)	118 (81.4%)	63 (45.0%)	78 (55.0%)
>=3(hazardous drinking)	160 (37.0%) (0.33, 0.42)	23 (16.0%)	74 (53.0%)	63 (44.0%)
Missing	8 (2.0%)	4 (3.0%)	3 (2.0%)	1 (1.0%)
**Do you take any kind of substances?**				
No	317 (74.0%)	110 (76.0%)	112 (80.0%)	95 (67.0%)
Yes	102 (24.0%) (0.19, 0.28)	32 (22.0%)	26 (19.0%)	44 (31.0%)
Prefer not to answer/ Missing	8 (2.0%)	3 (2.0%)	2/ (1.0%)	3 (2.0%)
**Have you ever used a needle to inject drugs?**				
No	419 (98.0%)	144 (99.0%)	136 (97.0%)	139 (98.0%)
Yes	5 (1.0%)	1 (1.0%)	2 (1.0%)	2 (1.0%)
Prefer not to answer	1 (<1.0%)	0 (0.0%)	1 (1.0%)	0 (0.0%)
Missing	2 (<1.0%)	0 (0.0%)	1 (1.0%)	1 (1.0%)
**Have you used a needle to inject drugs in the past month?**				
No	419 (98.0%)	144 (99.0%)	136 (97.0%)	139 (98.0%)
Yes	5 (1.0%)	1 (1.0%)	2 (1.0%)	2 (1.0%)
Prefer not to answer	1 (<1.0%)	0 (0.0%)	1 (1.0%)	0 (0.0%)
Missing	2 (<1.0%)	0 (0.0%)	1 (1.0%)	1 (1.0%)
**Polysubstance use and alcohol use**				
Both Alcohol and Substance Users	74 (17.3%)	15 (10.3%)	23 (16.4%)	36 (25.4%)
Poly Substance Users	54 (12.7%)	24(16.5%)	9 (6.4%)	21 (14.8%)

* Selective 95% CI on overall proportions.

Most women (74%) reported never using any illicit substances. Approximately one-quarter (24%, 95% CI [19%, 28%]) of participants reported taking any substance in the past month. Self-reported substance use was higher in Johannesburg (31%), followed by Harare and then Cape Town. As seen in Table 2, 17.3% (74) of women reported using both alcohol and a substance in the past month, while 12.7% (54) used more than one substance other than alcohol in the past month. Concurrent use of alcohol and substances was more common in Johannesburg, while polysubstance use was most common in Harare. Five women (1%) reported ever using a needle to inject substances across all sites.

The prevalence of use of different substances at baseline is shown in Table 3. The most used substances at baseline were cannabis (7%) followed by sedatives or sleeping pills (6.0%), and prescription drugs for non-prescription purposes (5.9%).

**Table 3:** Baseline prevalence of different substances.

Substance Use	n(%) (N = 427)	95% CI
**MARIJUANA) Cannabis, also called marijuana, pot, grass, dakka, dagga or hash**		
Never	382 (89.5)	[0.86, 0.92]
Ever	30 (7.0)	[0.05, 0.09]
Prefer not to answer/missing data	15 (3.5)	[0.02, 0.06]
**Cocaine, also called coke or crack**		
Never	392 (91.8)	[0.89, 0.94]
Ever	18 (4.2)	[0.03, 0.07]
Prefer not to answer/missing data	17(4.0)	[0.03, 0.06]
**Amphetamine-type stimulants, for example speed, diet pills, Tik/Crystal Meth or ecstasy**		
Never	397 (92.9)	[0.90, 0.95]
Ever	13 (3.1)	[0.02, 0.05]
Prefer not to answer/missing data	17 (4.0)	[0.03, 0.06]
**Inhalants, for example, nitrous, glue, petrol, and paint thinner.**		
Never	407 (95.3)	[0.93, 0.97]
Ever	5 (1.2)	[0.01, 0.03]
Prefer not to answer/missing date	15 (3.5)	[0.02, 0.06]
**Sedatives or sleeping pills, for example, serepax, rohypnol, quaaludes/mandrax**		
Never	388 (90.9)	[0.88, 0.93]
Ever	26 (6.0)	[0.04, 0.09]
Prefer not to answer/missing data	13 (3.1)	[0.02, 0.05]
**Hallucinogens, for example, LSD, acid, mushrooms, PCP, Special K**		
Never	394 (92.3)	[0.89, 0.94]
Ever	18 (4.2)	[0.03, 0.07]
Prefer not to answer/missing data	15 (3.5)	[0.02, 0.06]
**Opioids, for example, heroin (including nyaope/whoonga), morphine, methadone, etc**.		
Never	405 (94.9)	[0.92, 0.97]
Ever	7 (1.6)	[0.01, 0.03]
Prefer not to answer/missing data	15 (3.5)	[0.02, 0.06]
**Prescription drugs for non-prescription purposes, for example, codeine (including cough mixture), efavirenz, valium**		
Never	390 (91.3)	[0.88, 0.94]
Ever	25 (5.9)	[0.04, 0.09]
Prefer not to answer/missing data	12 (2.8)	[0.02, 0.05]
**Other**		
Never	283 (66.3)	[0.62, 0.71]
Ever	29 (6.8)	[0.05, 0.10]
Prefer not to answer/missing data	115 (26.9)	[0.23, 0.31]

### Hazardous drinking and substance use by study visit

As seen in Table 4, the proportion of young women reporting hazardous drinking remained relatively constant during the study, ranging from 33-37%. In contrast, the proportion of participants reporting substance use decreased over time, from 24.0% at baseline to 16.7% at study exit. The prevalence of substance use was highest at baseline (24%) and decreased at the week 13 visit (18.1%), the week 26 visit (16.2%), and at the week 52 visit (16.7%).

**Table 4:** Proportions of alcohol and substance use among PrEP acceptors by study visit.

Outcome	Week 13 (N = 370)	Week 26 (N = 364)	Week 52 (N = 347)
**AUDIT-C Score (Max score = 10)**			
<3 (Non-Hazardous Drinking)	224 (60.5%)	215 (59.1%)	208 (59.9%)
>=3(hazardous drinking)	122 (33%)	128 (35.1%)	123 (35.4%)
Prefer not to answer/ Missing	24 (6.5%)	21 (5.8%)	16 (4.6%)
**Ever consumed any substance**			
No	291 (78.7%)	295 (81.0%)	279 (80.4%)
Yes	67 (18.1%)	59 (16.2%)	58 (16.7%)
Prefer not to answer/ Missing	12 (3.2%)	10 (2.8%)	10 (2.9%)

### PREP adherence

PrEP adherence, defined as having intracellular TFV-DP concentration greater than 700 fmol/punch, decreased with study participation as seen in Table 5 below. PrEP adherence was highest at the week 13 visit (24.6%) and lowest at the study exit visit (8.7%). PrEP adherence was higher at the Johannesburg site compared to other sites with continued study participation (S1 Table).

**Table 5:** PrEP DBS Adherence by Visits.

	Week 13 (N = 370)	Week 26 (N = 364)	Week 52 (N = 347)
**PrEP DBS Drug Adherence**			
BLQ – 349 fmol/punch	193 (52.2%)	248 (68.1%)	294 (84.7%)
350 –700 fmol/punch	86 (23.2%)	40 (11.0%)	23 (6.6%)
>700 fmol/punch	91 (24.6%)	76 (20.9%)	30 (8.7%)

### Association between PrEP concentration, hazardous drinking, and substance use

Table 6 describes the proportion of women who were hazardous drinkers or substance users, who were low PrEP adheres with TFV-DP concentrations below 700 fmol/punch. As seen in Table 6, among women who were hazardous drinkers or substance users, the majority had low levels of TFV-DP and were low PrEP adherers. In addition, adherence to PrEP decreased with continued study participation. This is further shown in S1 Fig and S2 Fig, which show the TFV-DP concentration in hazardous drinkers and substance users by site.

**Table 6:** PrEP DBS Adherence by Hazardous drinkers & Substance Users by Visits.

	Week 13		Week 26		Week 52	
	Hazardous drinkers^1^ (n = 122)	Substance users^2^ (n = 67)	Hazardous drinkers^1^ (n = 128)	Substance users^2^ (n = 59)	Hazardous drinkers^1^ (n = 123)	Substance users^2^ (n = 58)
BLQ – 349 fmol/punch	60 (49.2%)	31 (46.3%)	89 (69.5%)	37 (62.7%)	102 (82.9%)	45 (77.6%)
350 –700 fmol/punch	32 (26.2%)	14 (20.9%)	12 (9.4%)	7 (11.9%)	8 (6.5%)	6 (10.3%)
>700 fmol/punch	30 (24.6%)	22 (32.8%)	27 (21.1%)	15 (25.4%)	13 (10.6%)	7 (12.1%)

^1^AUDIT-C score is >= 3.

^2^Any Substance user.

After adjusting for the study site, age, and education level, Hazardous drinking was associated with increased odds of low PrEP adherence, as shown in Table 7. Women classified as hazardous drinkers, with an AUDIT-C score of>=3, were 55.02% more likely to be low adherers compared to those with an AUDIT-C score <3 (aOR=1.55, 95% CI = 1.05- 2.29, p = 0.0265) (Table 7).

**Table 7:** Association between PrEP concentration and alcohol use.

Parameter	Odds Ratio^*^	95% CI	p-value	Unadjusted Odds Ratio^#^
Audit-C Score >=3	1.55	(1.05, 2.29)	0.0265	1.08
Audit-C Score <3	REF	REF	REF	1.00

* Odds ratio accounts for covariates: education level, pooled age group, and site.

The multinomial model has repeated measures of subjects over visits (week 13, 26, 52).

# Unadjusted Odds ratio does not account for covariates, education level, pooled age group, and site.

The multinomial model has repeated measures of subjects over visits (week 13, 26, 52).

Women who reported any substance use had 1.41 times increased odds of having low TFV-DP concentrations compared to women who did not report any substance use (aOR= 1.41aOR=1.41, 95% CI = 0.92- 2.16, p = 0.1145). However, this association was not statistically significant, as seen in Table 8.

**Table 8:** Association between PrEP concentration and substance use.

Parameter	Odds Ratio^*^	95% CI	p-value	Unadjusted Odds Ratio^#^
Substance Use	1.41	(0.92, 2.16)	0.1145	1.44
No Substance Use	REF	REF	REF	1.00

* Odds ratio accounts for covariates: education level, pooled age group, and site.

The multinomial model has repeated measures of subjects over visits (week 13, 26, 52).

# Unadjusted Odds ratio does not account for covariates, education level, pooled age group, and site.

The multinomial model has repeated measures of subjects over visits (week 13, 26, 52).

## Discussion

Our study found a comparable prevalence of alcohol use, hazardous drinking, and substance use among African AGYW. Hazardous drinking was associated with low adherence to PrEP. However, there was no association between substance use and low PrEP adherence. Hazardous drinking and substance use were highest at the beginning of the study and declined with continued study participation. The use of a needle to inject substances was rare. Cannabis was the most used substance, followed by sedatives and prescription drugs for non-prescription purposes.

Lifetime alcohol use in our study (57%) was comparable to that reported in the African Region, with the WHO reporting 55.5% of people between 15 and 24 years being current drinkers in 2016 [18]. Alcohol use among adolescents (10–19 years) is highest in Southern Africa, where our study is located, with an estimated prevalence of 40.82% in 2016 [24]. Hazardous alcohol use in South Africa, specifically, is considered an increasing public health concern as the country is reported to have the highest alcohol consumption in Africa, with the prevalence of harmful drinking being as high as 54.3% among female university students in Johannesburg. [17,18,35–37]. This is reflected in our results when considering the comparison of not only alcohol consumption but also hazardous drinking by site.

Despite alcohol consumption being predominantly in men, alcohol consumption and hazardous drinking in young women seems to be on the increase, characterized by periods of abstinence from alcohol followed by heavy episodic drinking, defined as having more than four drinks on one occasion [35,38,39]. Changes in gender norms and roles in society, partner influence, and the increased acceptability and accessibility of alcohol to women are thought to be drivers of increased alcohol consumption in this population [17,36,38].

Substance use in our study was lower (24%) than that found in other studies, with Olawole-Isaac et al reporting an overall prevalence of substance use of 37% in Southern Africa among adolescents in their systematic review [24]. However, the inclusion of caffeine inclusion use in this review could have resulted in an overestimation of substance use in this population, as caffeine is not commonly considered when speaking of illicit substances in the region. Substance use in the Zimbabwean cohort was higher than that found in other studies involving females within the Zimbabwean setting. A study conducted in 2019, to determine the prevalence of substance use among 10–24-year-old Zimbabwean youth, found that 13% of females within the population had used an illicit drug in the past three months [40]. Increased substance use among young Zimbabwean women has been attributed to adverse socio-economic conditions that have resulted in unemployment, poverty, and young women engaging in sex work and cross-border trading for income generation [41–44]. Increased exposure to substance use, coupled with the associated dangers that come with this line of work, necessitates the use of illicit substances to cope with various mental and physical pressures endured [41].

Cannabis was the most commonly used substance in this population. This is similar to reports by the United Nations Office on Drugs and Crime (UNODC) and WHO, who state that cannabis is the most frequently used substance globally, within the African region, and among young African women [17,45,46]. The prevalence of cannabis use in our study was higher than the reported population-level prevalence in SSA, with estimates of past cannabis use in the last 6months 6 months ranging from 4.5% in adolescents and 4.7% in adults in SSA, with the highest prevalence being among South African, Zambian and Zimbabwean adolescents [47]. The high prevalence of cannabis use has been attributed to the relative ease of access to the substance and differing risk perceptions associated with its use [48]. In addition, the use of cannabis is thought to be higher amongst vulnerable populations, such as those at higher risk of HIV acquisition who were enrolled in this study [47]. This may account for the higher prevalence that was reported.

Our finding that sedatives and prescription pills for non-prescription purposes were used more than amphetamine-type stimulants in this population was unexpected. Amphetamines are considered the second most common substance used in SSA [46]. The use of prescription medications, sedatives, and tranquilizers has been shown to be more common in women than in their male counterparts [45]. This is because women use these substances for various reasons, including weight control, exhaustion brought on by work and family responsibilities, and self-treatment of anxiety, depression, pain, and insomnia, conditions that are more common in women than men [45].

We found no association between ever substance use and low PrEP adherence. However, having an AUDIT-C score of>=3 was associated with low PrEP adherence. Our conflicting results are not uncommon. Many studies have looked at the association between alcohol and substance use with PrEP adherence and have yielded similar results [49]. Studies conducted in other high-incidence populations, such as gay and bisexual men who have sex with men and people who inject drugs have shown that alcohol and substance use were associated with low PrEP adherence while others have not, with more consistent evidence supporting the association being found in stimulant users specifically [28,29,49–51]. Such mixed findings have been attributed to methodological limitations associated with substance use research. These include variability in data collection methods and interpretation of substance use scores, the grouping of substance use into “never use” and “ever use” without taking into consideration the frequency and quantity of substances used, lack of collection of detailed substance use data including potential confounders as well as converting of TFV-DP levels from continuous data to categorical data [49]. Our analysis utilized similar methodological approaches used in previous studies, as it was a sub-analysis of a trial testing the efficacy of an intervention in PrEP users. It was not designed to focus on measuring substance use, and this may have resulted in lost sensitivity when conducting statistical analysis and an inability to detect potential or stronger relationships between the variables.

Despite such methodological limitations, we did find an association between hazardous drinking and low PrEP adherence. The use of PrEP among AGYW within southern and eastern Africa has been generally characterized by high initial uptake, followed by a gradual decline in adherence and persistence, similar to what was seen in our study [30,52–54]. Low PrEP adherence has been attributed to low-risk perception, pill burden, side effects, and lack of social support from family, partners, and the clinic due to multidose drug dispensing, resulting in infrequent clinic visits, amongst other reasons [52,55,56]. However, results from our study suggest that hazardous drinking may be an additional driver of low PrEP adherence in AGYW.

Adolescence, hazardous drinking, and substance use have been associated with increased sexual behaviors that increase the likelihood of HIV acquisition in SSA [17,27,57–59]. Considering this, more robust research designed to measure and analyze substance use in this population is needed to understand the relationship between hazardous drinking and substance use with PrEP adherence in this population, specifically in Southern Africa. Understanding if, and how, hazardous drinking and substance use are barriers to PrEP adherence in this context will assist in the development of comprehensive behavioral interventions and risk reduction tools targeted at adequately supporting PrEP uptake, adherence, and persistence in AGYW who would stand to benefit from this HIV prevention tool [50]. This is becoming essential as different biomedical HIV prevention modalities such as the dapivirine vaginal ring and long-acting injectables become available. Lastly, we recommend the integration of substance use and hazardous drinking screening and referral to treatment and harm reduction services within PrEP programs targeted towards AGYW. Screening can be done using validated tools by trained staff who are knowledgeable about both substance use and hazardous drinking [28].

This study has several limitations. Firstly, the generalizability of the study results may be limited as the study was conducted among a population of AGYW who were enrolled in a clinical trial and who were at high risk of HIV, which will likely not be representative of the larger population. Further, both hazardous drinking and substance use were determined through self-report and were therefore prone to underreporting due to the stigma and criminalization surrounding their use in our context. Future studies would benefit from more objective measures of hazardous drinking and substance use, such as reliable biomarkers. Lastly, the study was conducted at a time of low PrEP awareness due to a lack of PrEP guidelines. This could have impacted PrEP adherence levels observed. Despite these limitations, results from this study are supported by previous studies and hold practical implications for the delivery of PrEP in southern Africa and similar settings, especially given the dearth of information regarding substance use and hazardous drinking in these settings.

## Conclusion

In conclusion, hazardous drinking was associated with low PrEP adherence. Further research is required to determine the drivers and impact of substance use in AGYW at risk of HIV acquisition in the African context. This will allow for the implementation of both biomedical and behavioral interventions, aimed at preventing and treating AGYW substance users and informing HIV prevention programs, such as the rollout of PrEP targeted toward AGYW in SSA

## Supporting information

S1 Table Frequency of different drug use in the past month by visit. (DOCX)

S1 Fig TFV-DP concentration among participants for different levels of Audit-C score, by site and visit. (TIF)

S2 Fig TFV-DP concentration among participants for different levels of Drug use score, by site and visit. (TIF)
