# Supplementary material for: Association between substance use and PrEP adherence among adolescent girls and young women enrolled in an HIV prevention study in Southern Africa
Source: PLOS Glob Public Health. 2025 Jun 18;5(6):e0004750. doi: 10.1371/journal.pgph.0004750 (PMC12176117; doi:10.1371/journal.pgph.0004750)
Supplement: S1 Table — (DOCX) [file pgph.0004750.s001.docx]

**S1 Table: Frequency of different drug use in the past month by visit**

|  | **Baseline** | | **Week 13** | | **Week 26** | | **Week 52** | |
| --- | --- | --- | --- | --- | --- | --- | --- | --- |
|  | **Count** | **%** | **Count** | **%** | **Count** | **%** | **Count** | **%** |
| **(MARIJUANA) Cannabis, also called marijuana, pot, grass, dakka, dagga or hash** |  |  |  |  |  |  |  |  |
| Never | 382 | 89.5 | 344 | 90.8 | 341 | 91.4 | 340 | 92.4 |
| Less than monthly | 20 | 4.7 | 9 | 2.4 | 9 | 2.4 | 3 | 0.8 |
| Monthly | 2 | 0.5 | 1 | 0.3 | 1 | 0.3 | 4 | 1.1 |
| Weekly | 4 | 0.9 | 2 | 0.5 | 2 | 0.5 | . | . |
| Daily or almost daily | 4 | 0.9 | 7 | 1.8 | 6 | 1.6 | 7 | 1.9 |
| Prefer not to answer | 13 | 3.0 | 14 | 3.7 | 11 | 3.0 | 12 | 3.3 |
| Missing | 2 | 0.5 | 2 | 0.5 | 3 | 0.8 | 2 | 0.5 |
| **Cocaine, also called coke or crack** |  |  |  |  |  |  |  |  |
| Never | 392 | 91.8 | 347 | 91.6 | 343 | 92.0 | 345 | 93.7 |
| Less than monthly | 6 | 1.4 | 7 | 1.8 | 8 | 2.1 | 5 | 1.4 |
| Monthly | 5 | 1.2 | 3 | 0.8 | 3 | 0.8 | 2 | 0.5 |
| Weekly | 6 | 1.4 | 2 | 0.5 | 4 | 1.1 | 1 | 0.3 |
| Daily or almost daily | 1 | 0.2 | . | . | . | . | 1 | 0.3 |
| Prefer not to answer | 15 | 3.5 | 18 | 4.8 | 12 | 3.2 | 12 | 3.3 |
| Missing | 2 | 0.5 | 2 | 0.5 | 3 | 0.8 | 2 | 0.5 |
| **Amphetamine-type stimulants, for example speed, diet pills, Tik/Crystal Meth or ecstasy** |  |  |  |  |  |  |  |  |
| Never | 397 | 92.9 | 349 | 92.1 | 345 | 92.5 | 346 | 94.0 |
| Less than monthly | 8 | 1.9 | 6 | 1.6 | 9 | 2.4 | 5 | 1.4 |
| Monthly | 2 | 0.5 | . | . | 4 | 1.1 | 1 | 0.3 |
| Weekly | 1 | 0.2 | 4 | 1.1 | . | . | 1 | 0.3 |
| Daily or almost daily | 2 | 0.5 | 3 | 0.8 | . | . | . | . |
| Prefer not to answer | 15 | 3.5 | 15 | 3.9 | 12 | 3.2 | 13 | 3.5 |
| Missing | 2 | 0.5 | 2 | 0.5 | 3 | 0.8 | 2 | 0.5 |
| **Inhalants, for example nitrous, glue, petrol, paint thinner** |  |  |  |  |  |  |  |  |
| Never | 407 | 95.3 | 356 | 93.9 | 352 | 94.4 | 347 | 94.3 |
| Less than monthly | 1 | 0.2 | 3 | 0.8 | 5 | 1.3 | 6 | 1.6 |
| Monthly | . | . | 2 | 0.5 | 3 | 0.8 | 1 | 0.3 |
| Weekly | 2 | 0.5 | 1 | 0.3 | . | . | 1 | 0.3 |
| Daily or almost daily | 2 | 0.5 | . | . | 1 | 0.3 | . | . |
| Prefer not to answer | 13 | 3.0 | 15 | 4.0 | 9 | 2.4 | 11 | 3.0 |
| Missing | 2 | 0.5 | 2 | 0.5 | 3 | 0.8 | 2 | 0.5 |
| **Sedatives or sleeping pills, for example serepax, rohypnol, quaaludes/mandrax** |  |  |  |  |  |  |  |  |
| Never | 388 | 90.9 | 348 | 91.8 | 344 | 92.2 | 345 | 93.8 |
| Less than monthly | 15 | 3.5 | 11 | 2.9 | 9 | 2.4 | 4 | 1.1 |
| Monthly | 5 | 1.1 | 2 | 0.5 | 2 | 0.5 | . | . |
| Weekly | 4 | 0.9 | 1 | 0.3 | 2 | 0.5 | 5 | 1.4 |
| Daily or almost daily | 2 | 0.5 | . | . | 2 | 0.5 | 1 | 0.2 |
| Prefer not to answer | 11 | 2.6 | 15 | 4.0 | 11 | 3.0 | 11 | 3.0 |
| Missing | 2 | 0.5 | 2 | 0.5 | 3 | 0.8 | 2 | 0.5 |
| **Hallucinogens, for example LSD, acid, mushrooms, PCP, Special K** |  |  |  |  |  |  |  |  |
| Never | 394 | 92.3 | 350 | 92.4 | 350 | 93.8 | 348 | 94.5 |
| Less than monthly | 12 | 2.8 | 8 | 2.1 | 4 | 1.1 | 4 | 1.1 |
| Monthly | 1 | 0.2 | 4 | 1.1 | 3 | 0.8 | 1 | 0.3 |
| Weekly | 3 | 0.7 | . | . | 2 | 0.5 | 1 | 0.3 |
| Daily or almost daily | 2 | 0.5 | 1 | 0.2 | 1 | 0.3 | 1 | 0.3 |
| Prefer not to answer | 13 | 3.0 | 14 | 3.7 | 10 | 2.7 | 11 | 3.0 |
| Missing | 2 | 0.5 | 2 | 0.5 | 3 | 0.8 | 2 | 0.5 |
| **Opioids, for example heroin (including nyaope/whoonga), morphine, methadone, etc.** |  |  |  |  |  |  |  |  |
| Never | 405 | 94.9 | 357 | 94.2 | 352 | 94.4 | 350 | 95.0 |
| Less than monthly | 1 | 0.2 | 1 | 0.3 | 5 | 1.3 | 1 | 0.3 |
| Monthly | 3 | 0.7 | 2 | 0.5 | 2 | 0.5 | 1 | 0.3 |
| Weekly | 1 | 0.2 | 1 | 0.3 | . | . | 1 | 0.3 |
| Daily or almost daily | 2 | 0.5 | . | . | . | . | 1 | 0.3 |
| Prefer not to answer | 13 | 3.0 | 16 | 4.2 | 11 | 3.0 | 12 | 3.3 |
| Missing | 2 | 0.5 | 2 | 0.5 | 3 | 0.8 | 2 | 0.5 |
| **Prescription drugs for non-prescription purposes, for example codeine (including cough mixture), efavirenz, valium** |  |  |  |  |  |  |  |  |
| Never | 390 | 91.3 | 336 | 88.7 | 336 | 90.1 | 329 | 89.4 |
| Less than monthly | 19 | 4.5 | 13 | 3.4 | 12 | 3.2 | 14 | 3.8 |
| Monthly | 3 | 0.7 | 5 | 1.3 | 1 | 0.3 | 5 | 1.4 |
| Weekly | . | . | 8 | 2.1 | 4 | 1.1 | 4 | 1.1 |
| Daily or almost daily | 3 | 0.7 | 1 | 0.3 | 2 | 0.5 | 2 | 0.5 |
| Prefer not to answer | 10 | 2.3 | 14 | 3.7 | 15 | 4.0 | 12 | 3.3 |
| Missing | 2 | 0.5 | 2 | 0.5 | 3 | 0.8 | 2 | 0.5 |
| **Other** |  |  |  |  |  |  |  |  |
| Never | 283 | 66.3 | 256 | 67.6 | 261 | 70.0 | 262 | 71.2 |
| Less than monthly | 20 | 4.7 | 5 | 1.3 | 2 | 0.5 | 7 | 1.9 |
| Monthly | 4 | 0.9 | 3 | 0.8 | 1 | 0.3 | 4 | 1.1 |
| Weekly | 2 | 0.5 | 3 | 0.8 | 1 | 0.3 | 1 | 0.3 |
| Daily or almost daily | 3 | 0.7 | 4 | 1.1 | 4 | 1.1 | 2 | 0.5 |
| Prefer not to answer | 24 | 5.6 | 20 | 5.2 | 20 | 5.3 | 22 | 6.0 |
| Missing | 91 | 21.3 | 88 | 23.2 | 84 | 22.5 | 70 | 19.0 |
