# Supplementary figures and images for: Association between substance use and PrEP adherence among adolescent girls and young women enrolled in an HIV prevention study in Southern Africa
Source: PLOS Glob Public Health. 2025 Jun 18;5(6):e0004750. doi: 10.1371/journal.pgph.0004750 (PMC12176117; doi:10.1371/journal.pgph.0004750)

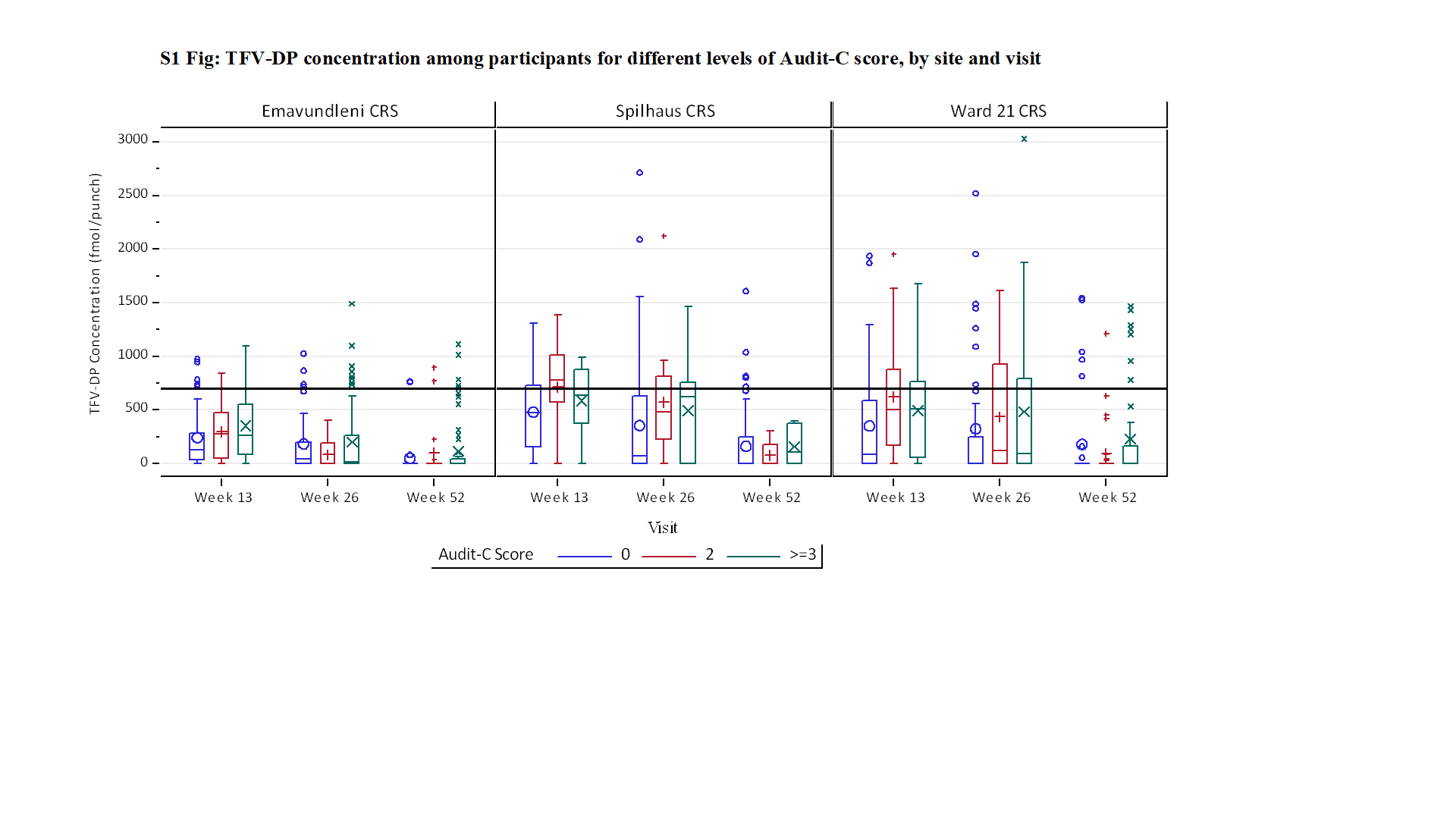

Supplement: S1 Fig — (TIF) [file pgph.0004750.s002.tif]

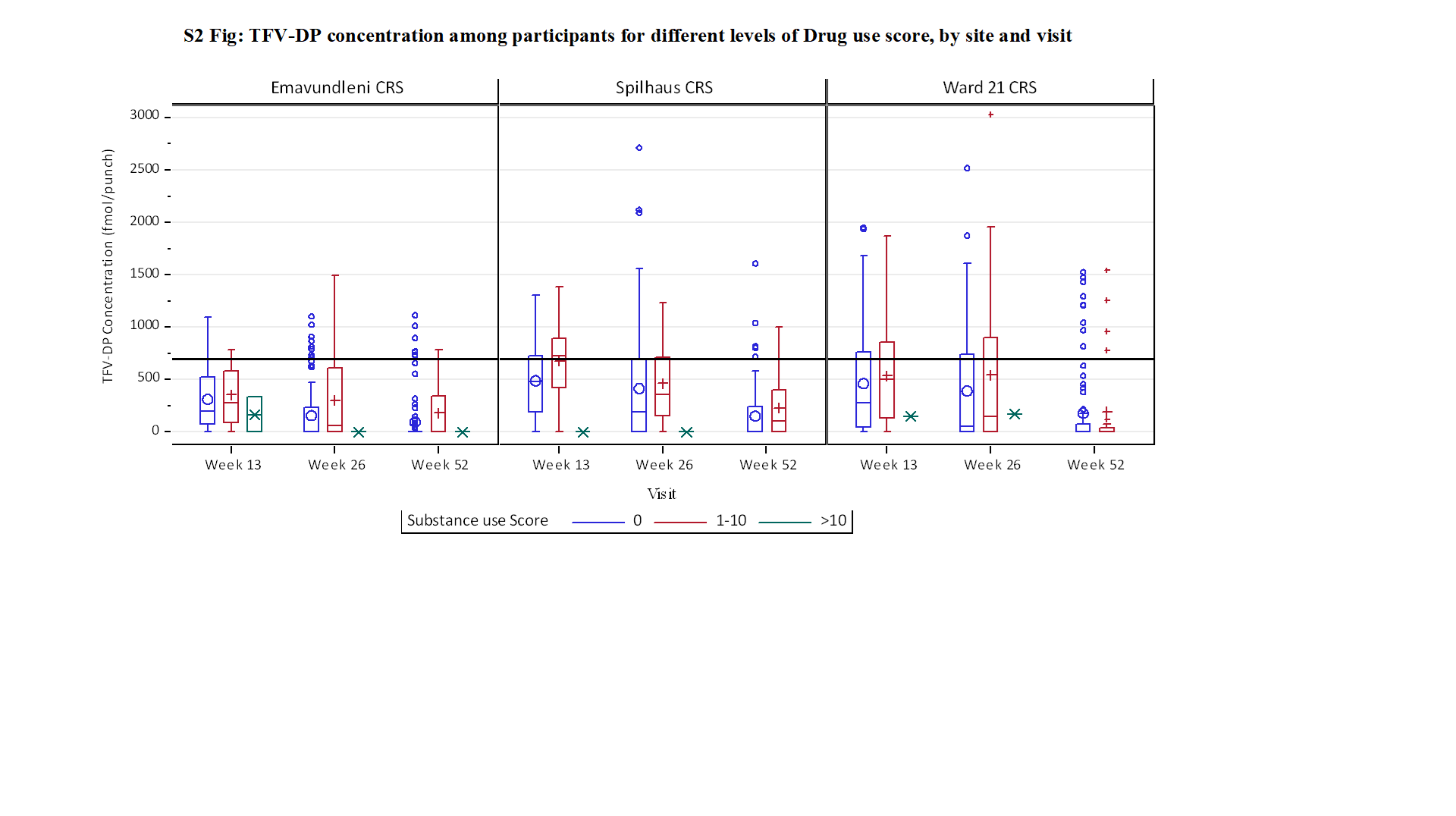

Supplement: S2 Fig — (TIF) [file pgph.0004750.s003.tif]
